# Intraperitoneal Haemorrhage Secondary to Rupture of Right Colic Artery Pseudoaneurysm: A Case Report and Literature Review

**DOI:** 10.7759/cureus.44634

**Published:** 2023-09-04

**Authors:** Prashanth B Chowdary, Gaurav Maheshwari, Maria Haynes, Iheoma Amaechi, Richard Dickson-Lowe

**Affiliations:** 1 General and Colorectal Surgery, Medway NHS Foundation Trust, Gillingham, GBR; 2 Pathology, Maidstone and Tunbridge Wells NHS Trust, Tunbridge Wells, GBR; 3 Interventional Radiology, Medway NHS Foundation Trust, Gillingham, GBR

**Keywords:** right colic artery, endovascular aneurysm repair, traumatic pseuoaneurysm, urgent laparotomy, multi-disciplinary team approach, haemoperitoneum, pseudo aneurysm

## Abstract

This article presents the case of a 58-year-old woman who presented feeling unwell with pain in the right upper abdomen for three days. She had a history of splenic infarcts, was on lifelong warfarin and had recently returned from a trip to Gambia. She was admitted to the hospital under suspicion of sepsis of unknown origin, and a CT scan later revealed haemoperitoneum along with a pseudoaneurysm of the right colic artery. After initially responding to resuscitation, the patient deteriorated haemodynamically, and a decision was made to perform a laparotomy, revealing a ruptured right colic artery pseudoaneurysm. In this article, the authors highlight the challenges of managing a complex unwell patient with a ruptured right colic artery pseudoaneurysm, emphasising the importance of a multi-disciplinary team approach and shared decision-making and reviewing the available literature.

## Introduction

Visceral artery pseudoaneurysm is a rare but potentially life-threatening condition that can result in significant morbidity and mortality if left untreated. It is often caused by trauma, infection, or inflammation and is characterised by the formation of a sac-like structure that communicates with an artery. These can rupture and cause haemorrhage into the surrounding tissues or body cavities, leading to shock, organ failure, and even death.

Diagnosing such aneurysms is challenging due to their nonspecific symptoms, and delay in diagnosis can have serious consequences. Early recognition and treatment are essential to prevent morbidity and mortality. Endovascular techniques have emerged as the preferred treatment method due to their minimally invasive nature and high success rates.

This article aims to review the current literature, including its epidemiology, aetiology, clinical presentation, diagnostic methods, and treatment options. We will discuss the role of imaging modalities in diagnosing visceral pseudoaneurysms and the various endovascular techniques used to treat this condition. We will also highlight the importance of a multidisciplinary approach in their management, involving interventional radiologists, surgeons, and critical care specialists. Overall, this article aims to increase awareness and understanding of such pseudoaneurysms among healthcare professionals and emphasise the importance of early recognition and prompt intervention to improve patient outcomes.

## Case presentation

A 58-year-old woman presented to the Accident and Emergency department with a history of feeling generally unwell and pain in the right upper aspect of the abdomen for three days. The pain was insidious in onset, gradually worsening to now become constant. She also had periods of feeling hot and cold and sweaty. She had a history of a one-week visit to Gambia and returned to the UK one week ago. During the visit, she had a trivial fall while walking up the stairs, which she felt did not warrant medical attention. On arrival in the UK, she visited another hospital with back pain. After examination, she underwent a computed tomography (CT) scan, which revealed no abnormality, and she was discharged with analgesia with a diagnosis of non-specific back pain. She had no headache, neck pain or stiffness, or symptoms suggestive of a respiratory or gastrointestinal tract infection. There was no history of fever or episodes of excessive bleeding.

Her medical history was significant for a right renal upper pole and multiple splenic infarcts three years prior, for which she was under the care of haematologists. The cause of the infarcts was not identified despite extensive investigations, and she had been prescribed lifelong warfarin. She was also hypertensive and had gastro-oesophageal reflux disease, anxiety disorder, and fibromyalgia. She had previously undergone lower segment Caesarean section and a laparotomy for adhesive small bowel obstruction. She was also on losartan, omeprazole and venlafaxine once a day and took atovaquone-proguanil as prophylaxis for malaria while in Gambia. 

On examination, she appeared pale and dehydrated while being alert and oriented to time, place, and person. Her pulse was 80 beats per minute (bpm) with a blood pressure of 116/67 mm Hg, her temperature was 37.1 degrees Celsius, and her oxygen saturation was 97% on room air. Systemic examination revealed no significant abnormality apart from mild tenderness with evidence of guarding in the right upper quadrant of the abdomen. Her blood tests revealed a haemoglobin of 105 g/l with a haematocrit of 31%. Her C-reactive protein was 71.8 mg/l, and her international normalised ratio (INR) was 9.3. The rest of her biochemistry panel was unremarkable. She was immediately administered intravenous (IV) vitamin K and started on IV levofloxacin. She was admitted under the care of the physicians for further evaluation with a suspicion of sepsis of unknown origin following foreign travel. Blood and urine cultures were performed, and a test for malarial parasites was also sent off, which eventually returned negative for Plasmodium falciparum and Plasmodium vivax.

The following day, she deteriorated further. Her pulse rate was 120 bpm, and her blood pressure had fallen to 96/50 mm Hg. A venous blood gas performed revealed haemoglobin of 72 g/l and lactate of 2.5 mmol/l, with the other parameters showing no significant abnormality. Her INR at this point was 1.4. Given her clinical appearance, she underwent an urgent chest, abdomen, and pelvis CT to identify the source of bleeding and/or sepsis. She continued to be resuscitated in the ward with IV fluids, blood products and antibiotics. The portal-venous phase CT scan revealed features of haemoperitoneum around the liver, spleen, the right paracolic gutter, lesser sac, and pelvis. There was also a 2.5 x 1.6 cm aneurysm in a branch of the right colic artery.

**Figure 1:**
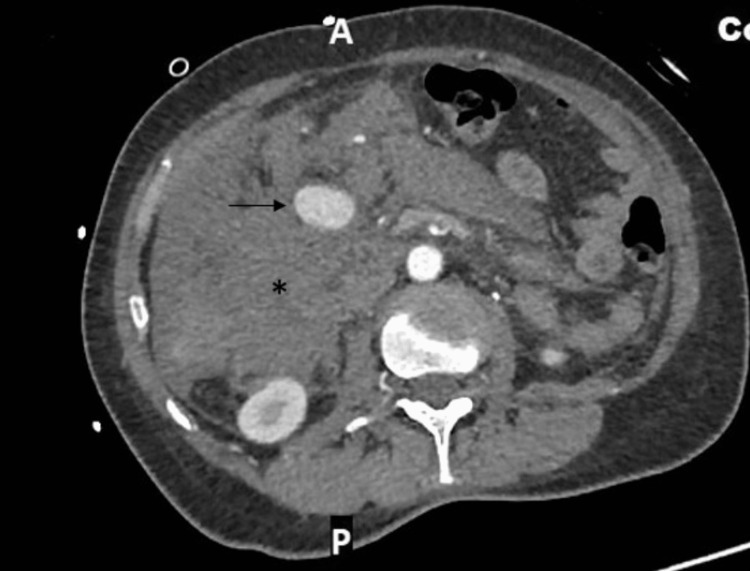
Arterial phase CT scan. Axial image demonstrating haemoperitoneum (*) and large pseudoaneurysm (thin arrow)

**Figure 2:**
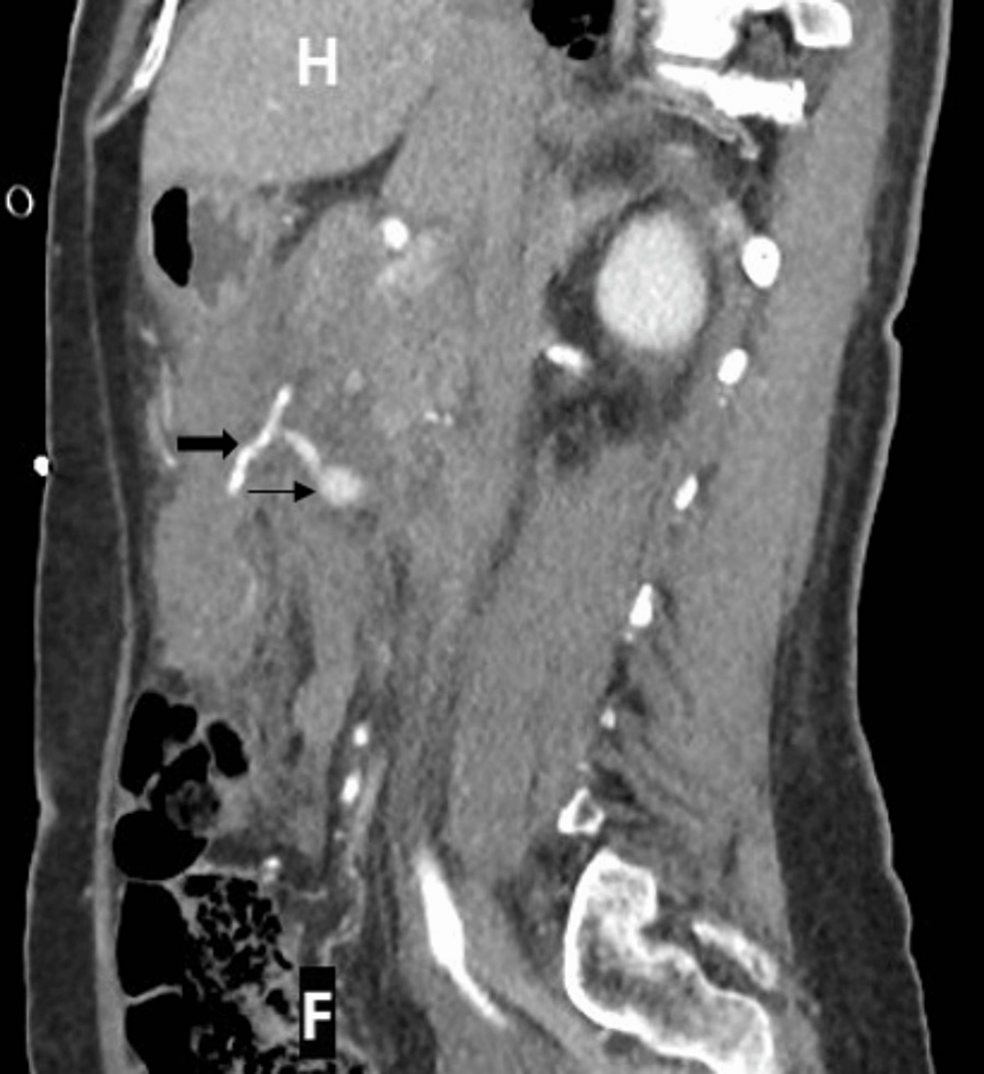
Arterial phase CT scan. Sagittal reformatted image demonstrating origin of pseudoaneurysm (thin arrow) from right colic branch of the superior mesenteric artery (thick arrow)

When the on-call surgical team reviewed her, she was diaphoretic and appeared unwell. She had abdominal distension and was guarding her abdomen, particularly in the right hypochondrium and lumbar region. However, there was no evidence of frank peritonism. As part of resuscitation, a Foley catheter was inserted, and advice was sought from the on-call haematologist about resuscitation using blood products, given current evidence of bleeding and a history of end-organ infarcts. She was also reviewed by the critical care out-reach nurses and the intensive care team, who were arranging for her transfer to the intensive care unit. The CT scan images were discussed with the on-call interventional radiologist, who suggested performing a CT mesenteric angiogram to characterise the aneurysm and demonstrate its anatomy. Since the patient had responded to resuscitation, a CT angiogram was performed that showed no increase in the size of the haemoperitoneum and no active contrast extravasation (Figure 1 and Figure 2). 

Unfortunately, following the CT scan, the patient deteriorated haemodynamically and was peritonitic. After a discussion between the on-call surgeon and the interventional radiologist, a decision was taken to perform a laparotomy as the patient was more unwell, the IR suite was in use for another emergency and endovascular treatment would require embolisation of the right colic artery branch, which would potentially render the bowel ischaemic eventually requiring a laparotomy. The patient consented to the procedure, including possible stoma formation and was quoted a mortality risk of 4.5% according to the National Emergency Laparotomy Audit. 

Intra-operatively, she was found to have massive haemoperitoneum with fresh blood in all four quadrants and a large haematoma at the root of the mesentery on the right side. The right colic artery branch pseudoaneurysm was identified and found to be very friable. The patient underwent a right hemicolectomy with ligation of the mesenteric vessels using multiple heavy absorbable sutures. The two stapled ends of the bowel were brought out through an incision in the right lower quadrant as a double barrel stoma. The abdomen was washed thoroughly and closed after placing two large drains in the pelvis and the right upper quadrant.

The patient recovered well post-operatively. She was on mechanical thromboprophylaxis for 72 hours following the procedure and restarted on prophylactic dalteparin on the fourth postoperative day. She was discharged on the 12th day following surgery with a plan to be reviewed in the haematology clinic about switching her back to an oral anticoagulant and in the surgical clinic to reverse her stoma.

**Figure 3:**
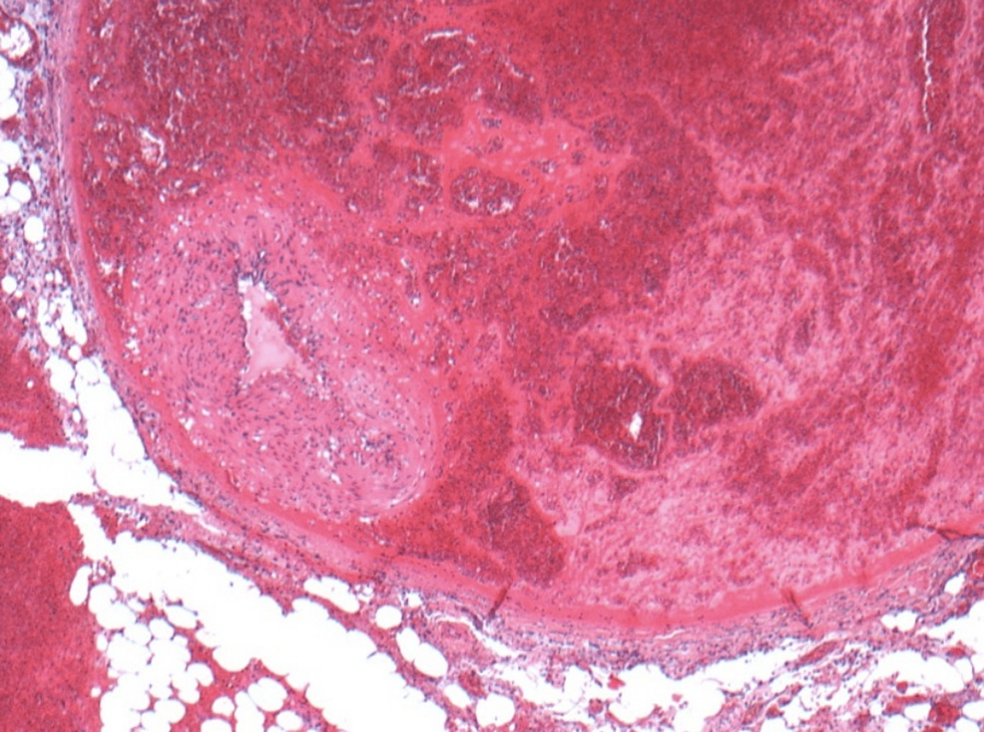
Hematoxylin and eosin (H&E) showing margination of the artery lumen and organising thrombus between muscle layers

**Figure 4:**
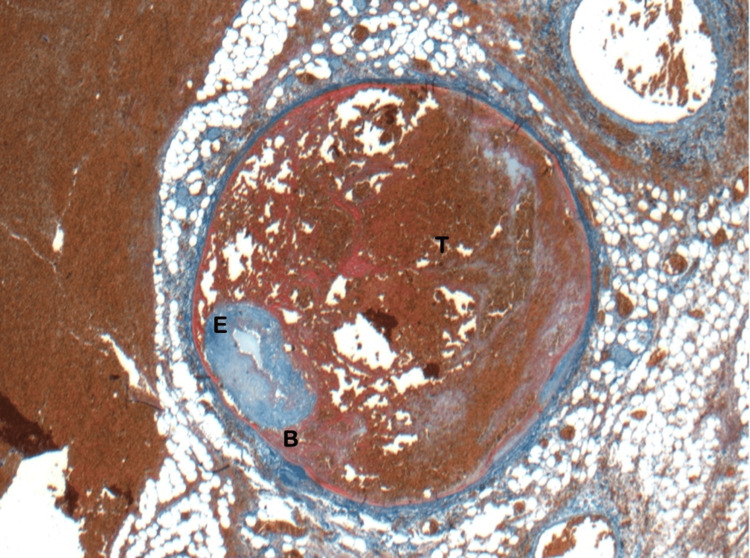
Martius Scarlet Blue (MSB): E- elastic fibres, B- blood between layers of vessel wall, T- Thrombus

The biopsy result of the specimen revealed a dissection and thrombus formation between the internal and external muscle layers of a medium-sized colic artery indicating a pseudoaneurysm (Figure 3 and Figure 4). There was also extensive interstitial haemorrhage throughout the mesentery. The rest of the bowel was unremarkable. 

## Discussion

The exact prevalence of visceral artery aneurysms might be challenging to evaluate due to their relative asymptomatic nature. From angiographic series and autopsy studies, the incidence has been estimated to be between 0.1% to 2% [1]. Among the earliest documented reports of a visceral artery aneurysm is the mention of one by Beaussier, who noted an aneurysm of the splenic artery while dissecting a 60-year-old female cadaver [2]. Literature review also provides us with Quincke’s triad, a triad of abdominal pain, obstructive jaundice, and upper gastrointestinal bleeding noted in hepatic artery aneurysms ruptured into the biliary system [3].

Aneurysms are generally defined as being true or false. True aneurysms have all three layers of the vessel wall intact. False aneurysms occur due to a tear in the wall, with a haematoma around the artery contained within a sac formed only by the media, adventitia, or surrounding tissues. The common causes for true aneurysms are atherosclerosis, medial degeneration/dysplasia, abdominal trauma, infection, inflammatory disease, connective tissue disorders such as Marfan syndrome, Ehlers-Danlos syndrome, Osler-Weber-Rendu disease, fibromuscular dysplasia, Kawasaki, hereditary hemorrhagic telangiectasia and hyper-flow conditions like portal hypertension and pregnancy. Pseudoaneurysms can occur due to iatrogenic causes like interventional, endoscopic, surgical procedures or secondary to trauma, inflammation, or infections [4-6].

Clinical presentation of pseudoaneurysms can vary and depend on their size and location. They can be asymptomatic and be picked up on clinical examination or radiological imaging. Patients can occasionally present with symptoms from the pseudoaneurysm, like pain or a palpable mass. In such scenarios, a palpable thrill or an audible bruit is evident. They can also present with symptoms secondary to the mass effect on surrounding structures like features of acute, acute-on-chronic or chronic ischaemia, neurological symptoms, or venous thrombosis. When these lesions rupture, the presentation can be more dramatic with patients exhibiting haemorrhagic shock, haematemesis, haematochezia, melena, or retroperitoneal haemorrhage [7-9]. Patients can sometimes present with features of the ’double-rupture’ phenomenon, described first by Bockerman in 1930. In this scenario, a rupture initially occurs into the lesser sac, leading to a transient tamponading effect until the haematoma finds its way through the foramen of Winslow into the greater sac or the peritoneal cavity, causing the patient to go into a state of severe haemorrhagic shock [10]. Nearly one in five patients with a pseudoaneurysm present as a clinical emergency with an overall mortality rate of 8.5% [11].

In the current era, the diagnosis is often made on imaging. Depending on the setting, either elective or emergency, the imaging modalities that can be used are ultrasound scans (contrast Doppler or contrast-enhanced), CT scans, magnetic resonance imaging, or digital subtraction angiography [12]. The ancient Chinese symbol of the “yin-and-yang” sign is a feature produced on a colour Doppler due to the swirling and turbulent movement of bidirectional blood flow [13]. The visceral arteries that commonly harbour these lesions are the splenic artery (60%), hepatic artery (20%), superior mesenteric artery (5%), and the gastric and gastroepiploic arteries (3%). Aneurysms of the colic branches are rare and account for 0.3% of superior mesenteric artery aneurysms [5,14,15].

Considering the high risk of morbidity and mortality associated with visceral pseudoaneurysms, they will all require treatment. The goal of therapy has to be to exclude the abnormal aneurysmal sac with the preservation of distal blood flow. However, this may not be possible in all cases. Surgical options involve an open or a keyhole approach to expose and exclude the abnormal sac. While this is an option in an elective or a haemodynamically stable patient, the approach must be one of damage control in a critically unwell patient. In the latter setting, often the only option is a damage control approach where the abnormal or bleeding vessel is ligated, the haematoma is evacuated, the abdominal cavity is washed, and the end organ is addressed appropriately [16,17]. The other option gaining popularity is the endovascular approach. Interventional radiologists can manage these lesions aggressively with a minimally invasive approach. Their treatment armamentarium continues to expand with such techniques as coil embolisation, placement of covered stents, plug deployment, glueing, and injection of endoluminal thrombin, polyvinyl alcohol, particles, or gel foam [18]. While the endovascular approach has the advantages of being minimally invasive, having a better quality of life in the peri-procedure period, and being associated with an overall reduced hospital stay, it may not always be possible to use this approach, especially in tortuous vessels. There is also the potential of end-organ ischaemia and necrosis to be considered. They are also associated with higher re-intervention rates, incomplete exclusion and might require repeat imaging [18-21].

Our patient presented with non-specific symptoms of feeling generally unwell for three days, hot and cold, dysuria, and right upper quadrant pain. As is often the case in such scenarios, a CT scan picked up the pseudoaneurysm. She was found to be too haemodynamically unstable, and there remained a concern of potential bowel ischaemia post-embolisation of the right colic artery, which precluded the endovascular approach. Whether the history of trivial trauma led to the formation of the pseudoaneurysm of the right colic artery is something that cannot be conclusively proven.

Idiopathic spontaneous intraperitoneal haemorrhage (ISIH), also previously called ‘abdominal apoplexy’, is an entity that refers to a spontaneous rupture of an abdominal blood vessel after exclusion of bleeding from an apparent aortic aneurysm, visceral malignancies, ectopic pregnancy and post-traumatic abdominal bleeding conditions. These patients are typically hypertensive and present in the fifth or sixth decades of life with a 2-3:1 male predominance [22,23]. Our patient could be labelled as having ISIH based on this criteria, but one should bear in mind that both terms were coined in the early 1900s [24,25], and with improvements in knowledge and technology, the number of truly ‘idiopathic’ conditions should reduce. 

## Conclusions

Patients with intra-peritoneal haemorrhage are critically unwell and require aggressive resuscitation, irrespective of the cause. We discuss a patient with a ruptured right colic artery branch pseudoaneurysm, her clinical presentation, imaging findings and management at our institute. These patients have a very high mortality risk and require prompt treatment. The aim should be to identify the cause of the bleeding and address it. The initial treatment option should be endovascular, and surgery should be considered only in cases where infrastructure is lacking, an endovascular approach has failed, or its risks outweigh the benefits. Surgical options will involve a laparotomy, excluding the pseudoaneurysm, ligating the culprit vessel, and addressing the end organ as appropriate. A multi-disciplinary team approach involving surgeons, diagnostic and interventional radiologists, haematologists, anaesthetists, and critical care teams is paramount in achieving a favourable outcome. 

## References

[1] Carr SC, Pearce WH, Vogelzang RL, McCarthy WJ, Nemcek AA, Yao JS (1996). Current management of visceral artery aneurysms. Surgery.

[2] Beaussier M (1770). Sur un anevrisme de l’artere splinque dont les parnis se sont ossifiees. J Med Toulose.

[3] Jamtani I, Nugroho A, Irfan W (2021). Revisiting Quincke’s triad: a case of idiopathic hepatic artery aneurysm presenting with obstructive jaundice. Ann Vasc Surg.

[4] Ferrero E, Viazzo A, Ferri M (2011). Management and urgent repair of ruptured visceral artery aneurysms. Ann Vasc Surg.

[5] Gehlen JM, Heeren PA, Verhagen PF, Peppelenbosch AG (2011). Visceral artery aneurysms. Vasc Endovascular Surg.

[6] Ikeda O, Tamura Y, Nakasone Y, Iryou Y, Yamashita Y (2008). Nonoperative management of unruptured visceral artery aneurysms: treatment by transcatheter coil embolization. J Vasc Surg.

[7] Yamakado K, Nakatsuka A, Tanaka N, Takano K, Matsumura K, Takeda K (2000). Transcatheter arterial embolization of ruptured pseudoaneurysms with coils and n-butyl cyanoacrylate. J Vasc Interv Radiol.

[8] Saad NE, Saad WE, Davies MG, Waldman DL, Fultz PJ, Rubens DJ (2005). Pseudoaneurysms and the role of minimally invasive techniques in their management. Radiographics.

[9] Okuno A, Miyazaki M, Ito H (2001). Nonsurgical management of ruptured pseudoaneurysm in patients with hepatobiliary pancreatic diseases. Am J Gastroenterol.

[10] Bizueto-Rosas H, Barajas-Colón JÁ, Delgadillo-de la O I, Malo-Martínez NP, Pérez-González HA, Hernández-Pérez NA (2016). [Multiple aneurysms splenic; surgical exclusion with conservation of the spleen]. Cir Cir.

[11] Ferrero E, Ferri M, Viazzo A (2011). Visceral artery aneurysms, an experience on 32 cases in a single center: treatment from surgery to multilayer stent. Ann Vasc Surg.

[12] Jesinger RA, Thoreson AA, Lamba R (2013). Abdominal and pelvic aneurysms and pseudoaneurysms: imaging review with clinical, radiologic, and treatment correlation. Radiographics.

[13] Deshpande S, Phatak S (2021). The yin-yang sign: footprint in diagnosis of pseudoaneurysm. J Datta Meghe Inst Med Sci Univ.

[14] Carter R, Gosney WG (1966). Abdominal apoplexy. Report of six cases and review of the literature. Am J Surg.

[15] Carr SR, Dinsmore RC, Wilkinson NW (2001). Idiopathic spontaneous intraperitoneal hemorrhage: a clinical update on abdominal apoplexy in the year 2001. Am Surg.

[16] Negmadjanov U, Ohanisian L, Rubay D, Hristov B, Belizon A (2019). Abdominal apoplexy: a case study of idiopathic spontaneous lesser sac hematoma. Cureus.

[17] Pulli R, Dorigo W, Troisi N, Pratesi G, Innocenti AA, Pratesi C (2008). Surgical treatment of visceral artery aneurysms: a 25-year experience. J Vasc Surg.

[18] Etezadi V, Gandhi RT, Benenati JF (2011). Endovascular treatment of visceral and renal artery aneurysms. J Vasc Interv Radiol.

[19] Cordova AC, Sumpio BE (2013). Visceral artery aneurysms and pseudoaneurysms—should they all be managed by endovascular techniques?. Ann Vasc Dis.

[20] Gabelmann A, Görich J, Merkle EM (2002). Endovascular treatment of visceral artery aneurysms. J Endovasc Ther.

[21] Uberoi R, Chung D (2011). Endovascular solutions for the management of visceral aneurysms. J Cardiovasc Surg (Torino).

[22] Cawyer JC, Stone CK (2011). Abdominal apoplexy: a case report and review. J Emerg Med.

[23] Watkins GL (1962). Abdominal apoplexy. Ann Surg.

[24] Barber MC (1909). Intra-abdominal haemorrhage associated with labour. Br Med J.

[25] Green WT, Powers JH (1931). Intra-abdominal apoplexy. Ann Surg.

